# Macrolide resistance via 23S rRNA gene mutation in *Corynebacterium ulcerans*

**DOI:** 10.1093/jac/dkag166

**Published:** 2026-05-19

**Authors:** Chiara Crestani, Sylvie Brémont, Marion Barbet, Kristina Museux, Edgar Badell, Sylvain Brisse

**Affiliations:** Biodiversity and Epidemiology of Bacterial Pathogens Unit, Institut Pasteur, Université Paris Cité, Paris, France; European Reference Laboratory for Public Health on Diphtheria and Pertussis (EURL-PH-DIPE), Institut Pasteur, Paris, France; European Reference Laboratory for Public Health on Diphtheria and Pertussis (EURL-PH-DIPE), Institut Pasteur, Paris, France; French National Reference Centre for Corynebacteria of the diphtheriae complex, Institut Pasteur, Paris, France; European Reference Laboratory for Public Health on Diphtheria and Pertussis (EURL-PH-DIPE), Institut Pasteur, Paris, France; French National Reference Centre for Corynebacteria of the diphtheriae complex, Institut Pasteur, Paris, France; Antech Diagnostics, Mars Petcare Science & Diagnostics, 10 rue du Saule Trapu, 91300 Massy, France; European Reference Laboratory for Public Health on Diphtheria and Pertussis (EURL-PH-DIPE), Institut Pasteur, Paris, France; French National Reference Centre for Corynebacteria of the diphtheriae complex, Institut Pasteur, Paris, France; Biodiversity and Epidemiology of Bacterial Pathogens Unit, Institut Pasteur, Université Paris Cité, Paris, France; European Reference Laboratory for Public Health on Diphtheria and Pertussis (EURL-PH-DIPE), Institut Pasteur, Paris, France; French National Reference Centre for Corynebacteria of the diphtheriae complex, Institut Pasteur, Paris, France

*Corynebacterium ulcerans* is a zoonotic pathogen that can cause diphtheria-like illness in humans, including respiratory infections, skin lesions and ulcers.^1–3^ Macrolide resistance in corynebacteria is so far known to be acquired through horizontal transfer of *erm* genes encoding rRNA methylases (notably *ermX*),^4^ and of genes coding for efflux pumps (*mef*).^4,5^ We describe a toxigenic (Elek test positive) *C. ulcerans* strain (ST326) from a cat showing macrolide resistance without identifiable resistance genes, but with a mutation in the gene coding for the 23S rRNA, the target of macrolide antimicrobial agents.

The strain was isolated from the nasal cavity of a 10-year-old FIV (Feline Immunodeficiency Virus)-positive neutered male European cat presenting chronic rhinitis and cough. Adopted from a shelter 5 years earlier, the cat had since then shown persistent respiratory symptoms and had possible occasional contact with farm animals (e.g. small ruminants). The owner reported that the cat underwent several antibiotic courses, although the full treatment history was incomplete due to management by multiple veterinary clinics. Under the last veterinarian’s care, two unsuccessful oral azithromycin courses were followed by a 15-day course of injectable marbofloxacin before the third sampling. Despite these treatments, *C. ulcerans* was isolated from three consecutive nasal swabs, confirming a monomicrobial infection with a bacterial load increasing from rare (<5 colonies; FRC2433, 7th February 2025) to moderate (5–20 colonies; FRC2469, 6th March 2025) and ultimately high (>20 colonies; FRC2490, 7th April 2025). Each isolate displayed high-level resistance to erythromycin, azithromycin, clindamycin, clarithromycin, and spiramycin (Table 1), whereas susceptibility to ciprofloxacin indicated no resistance to fluoroquinolones. Antibiograms were performed following EUCAST v16.0 (2026) breakpoints and methods.^6,7^ Shortly after the third sampling and the marbofloxacin course (26th April 2025), rhinoscopy was performed. Histopathological and cytological evaluations revealed marked exudative hyperplastic neutrophilic lymphoplasmacytic rhinitis. Although bacterial cultures from this specific rhinoscopy sampling were negative, maybe reflecting temporary bacterial suppression by the recent antimicrobial therapy, clinical respiratory symptoms persisted. The failure to eradicate the pathogen was confirmed four months later (August), when a follow-up swab yielded a polymicrobial culture containing moderate loads of both *C. ulcerans* and *Staphylococcus felis*.

**Table 1.** Results of antimicrobial susceptibility tests (disk diffusion diameters and minimum inhibitory concentrations) for the isolates in this study (NT: not tested)

Isolate	Isolation date	Erythromycin		Azithromycin		Clindamycin		Clarithromycin		Spiramycin	
		Diameter	MIC	Diameter	MIC	Diameter	MIC	Diameter	MIC	Diameter	MIC
FRC2433	07.02.25	6	>256	6	>256	6	>256	6	>256	13	NT
FRC2469	06.03.25	6	>256	6	>256	6	>256	6	>256	15	NT
FRC2490	07.04.25	6	>256	6	>256	6	>256	6	>256	15	NT
FRC1054	23.10.20	6	>256	6	>256	6	>256	6	>256	15	NT

Whole genome sequencing (short reads) revealed the absence of all known macrolide resistance determinants. Instead, a point mutation (A2257G) was identified in the 23S rRNA gene (corresponding to A2259G in *C. ulcerans* 809 reference genome and A2058G in *E. coli;* Figure 1). This substitution, previously associated with macrolide treatment failure in the Gram-negative bacteria *Bordetella pertussis,*^8,9^  *Helicobacter pylori,*^10^ and in *Mycoplasma pneumoniae,*^11^ alters a key target site in domain V of 23S rRNA, disrupting macrolide binding.

**Figure 1.**
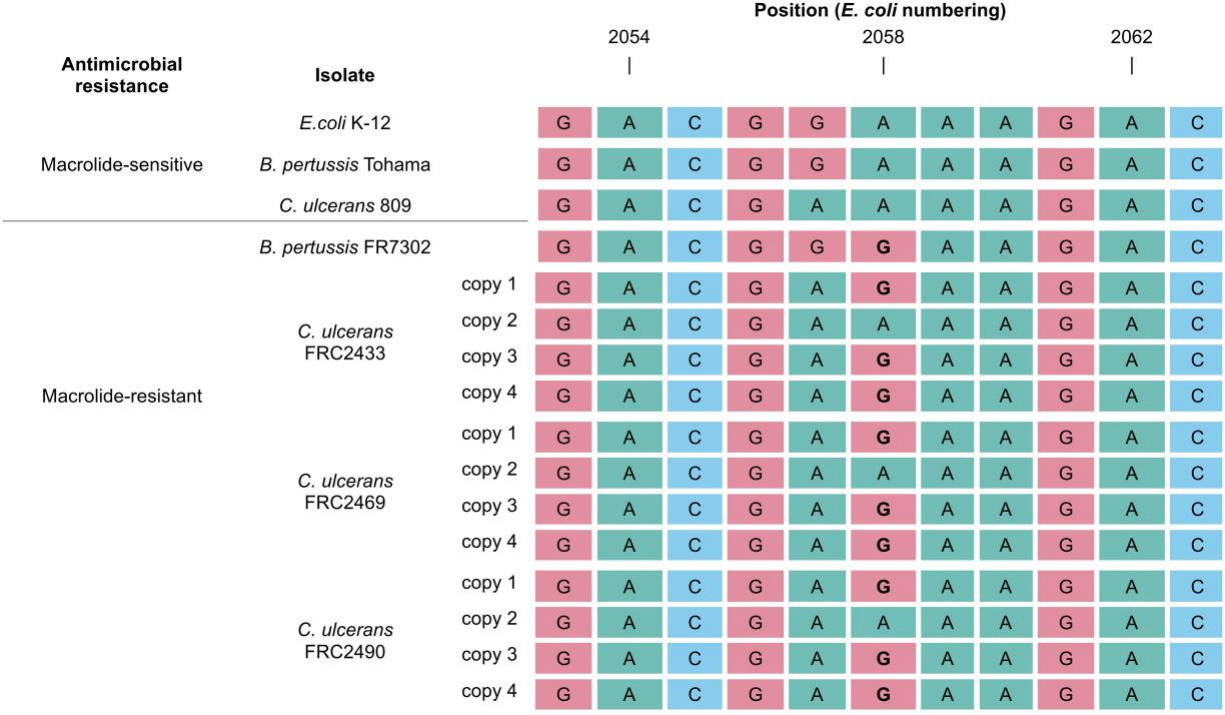
Multiple sequence alignment of a portion of the domain V of the 23S rRNA gene in genome assemblies from several isolates and bacterial species. The three sequences at the top include macrolide-sensitive isolates for reference. The sequences at the bottom include macrolide-resistant strains: *B. pertussis* FR7302 as reference for a highly resistant isolate carrying the mutation,^9^ and three *C. ulcerans* isolates from the case described here (FRC2433, FRC2469, FRC2490). All four copies of the gene are shown for each of the *C. ulcerans* isolates, highlighting how three out of four copies present the mutation.

The level of macrolide resistance depends on the proportion of rRNA gene copies carrying the mutation.^8^ As most bacteria harbour multiple 23S rRNA gene (*rrl*) copies, resistance becomes clinically significant only when most copies are mutated.^8^ Genome assembly algorithms for short-read sequences collapse these copies into a single locus, obscuring their individual states. Therefore, we applied long-read sequencing with Oxford Nanopore (R10.4.1 chemistry) to resolve all four *rrl* copies in *C. ulcerans*. In the three isolates, three of the four 23S rRNA gene copies carried the A2257G mutation, consistent with the observed high-level macrolide resistance. The cat’s prolonged exposure to macrolides, likely at sub-optimal dosage due to poor tolerance of oral therapy, probably selected for this mutation.

Re-examination of the collection of the French NRC for diphtheria revealed another *C. ulcerans* isolate (FRC1054, from a cutaneous infection in a cat in 2020, and an antibiogram profile similar to the isolates in this study; Table 1) with unexplained macrolide resistance (corresponding to the macrolide-resistant isolate from Berger *et al*.^6^). This strain also carried the same A-to-G substitution (Illumina sequencing; all *rrl* copies conflated). Although prior antibiotic exposure was unknown, its distinct sequence type (ST339) indicates independent emergence of this mutation in two different phylogenetic lineages.

This is the first report, to our knowledge, of macrolide resistance associated with a chromosomal mutation in corynebacteria. Although this association does not provide evidence for causality, the fact that this mutation is observed at the same position in the two *C. ulcerans* macrolide-resistant strains with no *ermX* or *mef* genes, and that this position corresponds to the one previously associated with macrolide resistance in several other bacterial species, strongly suggests causality. This case carries important implications. The emergence of macrolide resistance in a toxigenic *C. ulcerans* strain raises concern for treatment efficacy, since macrolides are first-line agents for diphtheria management in penicillin-allergic human patients. The observed resistance phenotype in veterinary practice severely limits therapeutic options, as the unofficial treatment of choice for corynebacterial infections is macrolides (e.g. azithromycin).^3^ This is particularly problematic in settings involving immunocompromised animals with persistent infections, as illustrated in the present case. The chromosomal location of the 23S rRNA mutation implies its vertical inheritance within lineages where it arises, although its long-term prevalence will depend on the balance between any associated fitness cost and the selective pressure exerted by macrolide exposure. Official, evidence-based veterinary guidelines for diphtheria management are currently lacking in our country and others. Such guidelines would be useful to provide standardized therapeutic recommendations and to inform veterinarians on infection control measures and mitigation of the zoonotic risk. More broadly, this report illustrates the value of a One Health approach of diphtheria surveillance, making a compelling case for the inclusion of veterinary isolates of the CdSC to capture the full spectrum of antimicrobial resistance, toxigenicity and transmission dynamics across reservoir animal species and humans.

In conclusion, we report a 23S rRNA gene A2257G mutation associated with resistance mechanism to macrolides in *C. ulcerans*. This expands our understanding of resistance to this important antimicrobial class in the genus *Corynebacterium* and stresses the importance of integrating genomic analysis into surveillance workflows.

## Data Availability

Long-read genomic sequences were deposited on the European Nucleotide Archive under project number PRJEB107096 (accession numbers ERS28579838, ERS28579839, ERS28579840).
